# The Abundance and Structure of Deadwood: A Comparison of Mixed and Thinned Chinese Fir Plantations

**DOI:** 10.3389/fpls.2021.614695

**Published:** 2021-03-03

**Authors:** Yuanfa Li, Muxuan Li, Xian Li, Zhilong Liu, Angang Ming, Huangxu Lan, Shaoming Ye

**Affiliations:** ^1^College of Forestry, Guangxi Key Laboratory of Forest Ecology and Conservation, Guangxi University, Nanning, China; ^2^Department of Renewable Resources, University of Alberta, Edmonton, AB, Canada; ^3^Experimental Center of Tropical Forestry, Chinese Academy of Forestry, Pingxiang, China; ^4^Guangxi Youyiguan Forest Ecosystem Research Station, Pingxiang, China

**Keywords:** biodiversity, Chinese fir, competition, distribution pattern, sustainable forest management, thinning

## Abstract

The sustainability of coniferous monoculture plantations is facing challenges with respect to yields, ecology, and biodiversity. Conversion of monocultural coniferous plantations into mixed stands using thinning or direct mixed planting is widely considered to be a key strategy for overcoming these challenges and transforming the characteristics of plantations on a regional scale. Substantial amounts of deadwood may be produced in mixed forests (MFs); this material is important for evaluating and modifying forest management methods, understanding the dynamics of forest stands, and achieving biodiversity conservation. We assessed the quantitative characters and diameter distributions of deadwood in mixed and thinned Chinese fir [*Cunninghamia lanceolata* (Lamb.) Hook.] forests over one rotation. We used the *g*(*r*) function and spatial parameters to analyze the spatial structure of deadwood, and used logistic regression and Hegyi’s competition index (HCI) to explore competition and mortality. Our results indicate that: (1) Chinese fir dominated in all groups of deadwood (snags, broken wood, and fallen wood), and the abundance, volume, and mortality rates of deadwood were much lower in the thinning forest compared to the MF. (2) Later coming populations (LCPs) comprised the majority of the small diameter classes in the thinning forest, but only accounted for a small proportion of the MF. (3) Broken wood in the thinning forest was randomly distributed, while the other types of deadwood were clustered at most spatial scales. In contrast, the spatial patterns in the MF were random at most spatial scales. (4) Total deadwood in both stands was in a status of intermediate and was randomly surrounded by its four nearest neighbors. All types of deadwood were highly mixed in the thinning forest and moderately mixed in the MF. Our case study suggests that thinning and mixing result in different stand development processes and thus influence the type, amount, and structure of deadwood. Thinning significantly reduces competition, which is the main driver of tree mortality. Converting pure Chinese fir plantations into mixed stands by thinning should be taken in future. Understanding tree mortality after conversion is essential to select appropriate silvicultural treatments and achieve ultimately sustainable forest management.

## Introduction

The dynamics of tree mortality are an important characteristic of forest stands (Harvey and Brais, 2007; Gray and He, 2009; Laarmann et al., 2009; Moussaoui et al., 2020), with implications for forest ecosystem functions (i.e., material cycling and energy flows), forest resilience and stability (Siitonen et al., 2000; Bölöni et al., 2017; Oettel et al., 2020; Zhang et al., 2020). Different types of deadwood are formed following tree death, including standing dead trees (snags), fallen wood (logs), and broken wood (Girona et al., 2019). Natural forests, especially those in temperate regions, have an abundance of deadwood that supports the growth and vitality of retained and regenerating trees (Martin et al., 2020). Deadwood also provides resources, such as food and habitat, for small animals (e.g., birds, insects, and arthropods) as well as saprophytes and parasitic plants (e.g., lichens, mosses, fungi, and mushrooms) (Franklin et al., 1987; Dittrich et al., 2014; Kim et al., 2020; Monaco et al., 2020; Oettel et al., 2020), and has been analyzed in studies of forest carbon stocks, soil development, and climate change (e.g., Bölöni et al., 2017; Farahat et al., 2017; Zhang et al., 2020). During the past 40 years, mechanisms of deadwood formation in natural forests have been well-expounded (e.g., endogenous *vs.* exogenous interference, natural vs. human disturbance, biotic vs. abiotic factors, and density-dependence) (Franklin et al., 2007; Laarmann et al., 2009; Iida et al., 2014; Zhang et al., 2017; Kweon and Comeau, 2019). Conversely, plantations are often intensively managed, and are characterized by monocultures, short harvesting cycles, and clear-cutting. Many stands are harvested before substantial divergence in tree size and senescence occurs, and the abundance and species richness of deadwood are typically low (Siitonen et al., 2000; Franklin et al., 2007; Zhao et al., 2007). At the same time, data on deadwood in plantations are sparse, and formation processes are consequently poorly understood.

Globally, the continuous expansion of plantation and crop areas (e.g., *Pinus*, Taxodiaceae, and *Eucalyptus*, which are widely planted in forested regions of southern China, and *Larix* and *Populus*, which are abundant in the northeast), has had numerous ecological impacts [e.g., habitat loss and fragmentation, biodiversity loss, intensification of pests and diseases, low resilience, and poor recovery after disturbance (Sheng and Xue, 1992; Li, 2004; Zhu et al., 2010; Lu et al., 2018)], and has also led to environmental degradation in the form of soil erosion, landslides, and decreased soil fertility and water holding capacity (Li, 2004; Lu et al., 2018). Thinning and underplanting are widely used to transform plantations into uneven-aged mixed forests (MFs) in an effort to eliminate or mitigate such impacts, and to alter the characteristics of plantations at the regional scale (e.g., Parker et al., 2001; Zhu et al., 2010; Otto et al., 2012; Lu et al., 2018; Zhang et al., 2019; Ming et al., 2020). Mixed stands may also be planted from scratch (e.g., Piotto et al., 2004; Li et al., 2020). Additional measures, such as extending rotation times or closing hillsides to facilitate afforestation, are sometimes used in conjunction with natural processes to promote stand succession (Li, 2004; Cai et al., 2007; Hilmers et al., 2020). Competition between trees and consequent mortality are inevitable in stand cultivation and natural conditions (Girona et al., 2017). However, most studies focus on short term changes in species diversity, growth, stocking rates, carbon storage, soil nutrients, and microorganisms following conversion or mixed planting (e.g., Sun et al., 2015; Zhang et al., 2019), ignoring the influence of management measures on the formation of deadwood.

Chinese fir is a fast-growing tree that produces high-quality timber. It is the most common plantation species in the subtropics, accounting for 24% of the plantation area in China and 6.1% of the global plantation area (Feng et al., 1988; Liu et al., 2018). The cultivation of Chinese fir goes back thousands of years in China. Chinese fir plantations play an important role in climate change, regional ecological and environmental protection, employment, economic income, and timber supply (Zhang et al., 2017, 2019, 2020; Li et al., 2020); however, they are also associated with increasingly concerning declines in soil health and tree growth (Chen et al., 1990; Sheng and Xue, 1992; Wang and Wang, 2008; Liu et al., 2018). We have been conducting field experiments in major Chinese fir-producing areas since the early 1990s, with the objectives of changing unsustainable planting and harvesting methods and developing ecologically sound practices that promote species diversity, yield multiple benefits, and produce a range of products. Some of these stands have reached the full rotation period of 25 years (Li et al., 2020). Here we explore the quantity and structural characteristics of deadwood in thinned and mixed Chinese fir plantations at the stand level, to better understand the dynamics of deadwood formation in plantations. This is particularly important for the evaluation and modification of forest management practices, as well as for understanding stand dynamics and protecting biodiversity.

Thinning decreases tree density and reduces competitive pressure among neighbors (Zhu et al., 2010; Lu et al., 2018; Li et al., 2020). We hypothesized that the quantity of deadwood would be lower in thinned stands than in mixed stands, and that surviving planted trees in thinned stands would be larger than those in mixed stands (Hypothesis 1). Thinning releases resources and promotes the regeneration and growth of other species (Franklin et al., 2007; Otto et al., 2012; Li et al., 2020), which may in turn promote greater diversity, whereas MFs exhibit a high degree of species mixture by design (Li et al., 2020). Therefore, we assumed that the species richness of deadwood in two stands would be similar (Hypothesis 2). Because planting is uniform, and deadwood likely originates primarily from planted trees (although some may represent unplanted colonizing species), we assumed that the distribution patterns of deadwood would be uniform in both stand types (Hypothesis 3).

## Materials and Methods

### Study Area and Experimental Design

**FIGURE 1:**
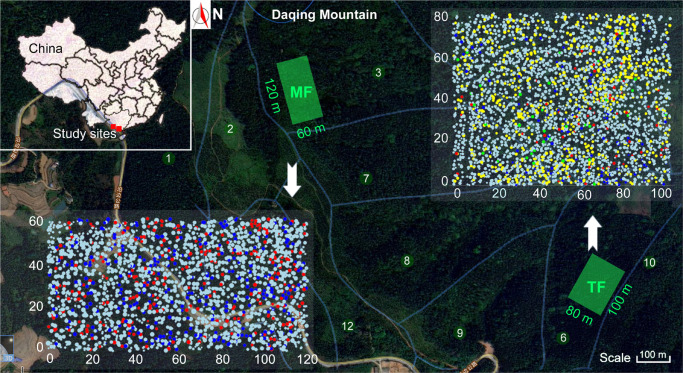
The study plots, located on the mid-slope of Daqing Mountain. Green squares indicate the location and size of plots, and Arabic numerals indicate compartment number. The green, red, blue, and yellow dots in each quadrat represent broken wood, live trees, snags, and stumps, respectively.

Our study field was located on the mid-slope of Daqing Mountain, the highest peak in Pingxiang City and Longzhou County (altitude = 1,045.9 m a.s.l., local relief = 916 m) (Figure 1). The Daqing Mountain was located at the Qingshan forestry farm at the Experimental Center of Tropical Forestry of the Chinese Academy of Forestry and has a typical south Asian tropical climate. This region has abundant rainfall and heat resources. Its average annual temperature is between 19.5 and 21.5°C and average annual rainfall is around 1,200–1,500 mm. The wet season (April–September) is in spring and summer, and the dry season (October–March) is in autumn and winter (Cai et al., 2007; Sun et al., 2015; Li et al., 2020). The soil is a weakly acidic mountain red soil (pH 4.8–5.5), with a thickness of more than 80 cm (Sun et al., 2015). Its primary vegetation was mainly composed of seasonal rain forest and rainforest, and had been destroyed for a long time (Li et al., 2020).

We have been conducting a variety of silvicultural studies in plantations in and around the region since 1979 in a range of stand types, including fast-growing, high-yield forests, short-cycle rotation forests, semi-natural forests, MFs comprising both native and exotic species, and MFs of fast-growing, economically valuable species (Ming et al., 2014, 2018, 2020; Sun et al., 2015; Li et al., 2020). As part of this work, a MF of Chinese fir [*Cunninghamia Lanceolata* (Lamb.) Hook.] and *Michelia macclurei* Dandy was planted in 1991 in the third compartment (106° 42′ 16″ E, 22° 18′ 9″ N) at a ratio of 6:1 *C*. *lanceolata*: *M. macclurei*. Following random mixing, trees were uniformly planted at an initial density of 2,000 plants/ha. The survival rate was 87% after 2 years of management. A pure Chinese fir forest was also established in a neighboring compartment (106° 42′ 48″ E, 22° 17′ 44″ N), and during the same period, with similar density and spatial patterns and a survival rate of approximately 87%. Thinning was conducted in this stand (hereafter referred to as TF) in 2008, where 26% of individuals were removed. Trees with a diameter at breast height (DBH, *d*_1.3_) ≥ 9 cm were harvested at random by experienced forestry engineers, and logs were removed from the forest for commercial sale (Li et al., 2020). Since then, no major natural disasters or human disturbances have occurred in either stand and both have developed into multi-species forests.

### Plot Establishment

A standard permanent plot was established in each of the study stands. The MF plot was 120 m × 60 m and the TF plot was 100 m × 80 m (green squares in Figure 1). We investigated the terrain and area of each sub-compartment in the field prior to selecting specific locations for plots. Next, we used a total station (NTS-372R_10_; South Surveying and Mapping Company, Guangzhou, China) to locate the boundaries of each plot and recorded the three-dimensional coordinates of all individuals with a DBH > 1 cm, as well as stumps in the TF (yellow dots in Figure 1). Then, we classified all trees within each plot as either live trees or deadwood. Live trees included live later coming populations (LCPs) and live planted Chinese fir and *M. macclurei*. Deadwood included snags, fallen wood, and broken wood. We measured the DBH (*d*_1.3_), height (*h*) and length (*l*), and crown width of all live trees, and graded the decay (five grades in all) and recorded the central diameter (*d*_½_) of fallen wood. In addition, we tagged live trees and identified each individual to species. We collected data on a total of 1,909 live trees (655 Chinese fir, 182 *M. macclurei*, and 1,072 LCPs) and 558 pieces of deadwood in the MF. In the TF, we surveyed 3,084 live trees (654 Chinese fir and 2,430 LCPs), 639 stumps, and 218 pieces of deadwood. Additional details about species composition and characteristics are available in Li et al. (2020).

### Data Analysis

Quantitative characteristics of interest of the forest stands included mean or count values of DBH, height, age, and volume, etc. Structure refers to the distribution of these variables and can be represented numerically and as correlations between factors, such as tree height distribution and DBH class. Structure can also refer to other spatial properties, e.g., size differentiation, species segregation and point patterns (Li et al., 2012, 2020; Hui et al., 2019). The analyses of quantitative characteristics and structure in this study were conducted using R software (version 3.62; R Core Team, 2019).

#### Deadwood Quantity

We counted the number of each type of deadwood and calculated the proportion of the total accounted for by Chinese fir. We determined the volume (m^3^) of all individuals of Chinese fir based on the two-way volume table for local tree species (Anonymous, 1986). For species not included in the table, we calculated single tree volume using the formula: V=π40000d21.3×(h+3)×f∂ (Equation 1). For broken wood, we used the formula: V=π40000×d212×l (Equation 2). In these equations, *l* (m) and *h* (m) represent the length and height of trees, 3 is a constant, and *f*_*∂*_ is a reduction factor; *f*_*∂*_ = 0.4 for conifers and *f*_*∂*_ = 0.2 for broadleaved trees (Meng, 2006). In addition, we used: $\bar{d}=\frac{1}{N}\sum_{i=1}^{N}d_{i}$ (Equation 3) to calculate the mean DBH for each species/group of live trees and each category of deadwood. We used Wilkinson and Cleveland dots in the *ggplot2* R package (Wickham, 2016) to derive mean values.

#### Deadwood Structure

Deadwood structure was assessed based on the DBH distribution, point patterns, and structural diversity (SD). We used the *hist* function to generate histograms of DBH distributions for deadwood, snags, fallen wood, and broken wood, and used the *nls* and *ks*.*test* functions to fit curves to the histograms. We used the pair correlation function (PCF) *g*(*r*) to analyze point patterns. PCF is one of the most and powerful and widely used methods for analyzing tree point patterns; in addition to effectively resolving the issue of scale dependence in distribution patterns and eliminating cumulative errors resulting from semicircles in the statistical process (Getzin et al., 2006, 2008; Gray and He, 2009). According to stand age and tree size, we set the maximum radius (*r*) of the circle to half the distance of the short side of the plot (*r* = 60/2 m). We used complete spatial randomness (CSR) as a null model, the “best” option for edge correction, and 999 Monte Carlo simulations (MCS) at each scale. We then tested the goodness of fit relative to an envelope bounded by the 2.5% and 97.5% values from the MCS. Analysis was conducted using the *spatstat* R package (Baddeley et al., 2015).

We used a number of parameters to analyze SD, including the uniform angle index (W), mingling (M), and dominance (U), based on the spatial relationships among the four nearest neighbors and a reference tree *i* (also called the “structure unit”) (Laarmann et al., 2009; Li et al., 2012, 2020; Hui, 2013; Hui et al., 2019). The dataset was first divided into live LCPs, live planted trees, snags, fallen wood, and broken wood. We treated the area within 5 m of the plot perimeter as the buffer area; the remaining area was considered as the core area. Trees in the buffer area were only included as neighbors in structural assessments. Finally, we calculated the value of each parameter for all trees in the core area, and the arithmetic mean for each group. Values of W, M, and U range between 0 and 1. The value of M is proportional to the degree of species mixing, while the value of U is inversely proportional to the degree of dominance. Low values of W indicate a uniform distribution, while moderate values indicate a random distribution, and high values aggregation (Hui, 2013; Li et al., 2017, 2020; Hui et al., 2019).

#### Causal Analysis of Deadwood

Tree condition was analyzed as a binary variable (live = 0, dead = 1). Tree mortality rates can be derived using a logistic regression function, $p=\frac{1}{1+e^{-\eta }}$ (Equation 4), where $\eta =\text{logit}\left(p\right)=ln\left(\frac{p}{1-p}\right)=\beta_{0}+\beta_{1}\times d_{1.3}$ (Equation 5); this method has been widely used to determine the causes of tree mortality in natural forests (e.g., Laarmann et al., 2009; Hurst et al., 2012; Wu et al., 2017; Kweon and Comeau, 2019). It is also used to predict tree mortality and survival rates in plantations (e.g., Zhao et al., 2007; Bladon et al., 2008; Zhang et al., 2017, 2020). We analyzed relationships between deadwood and live trees using the *roc* and *AUC* functions in the *pROC* R package, to calculate the area under the receiver operating characteristic curve (AUC) (Robin et al., 2011). AUC values range between 0 and 1, and we defined goodness of fit as follows: 0.8–1.0 = excellent, 0.6–0.8 = good, 0.4–0.6 = poor, 0.0–0.4 = fail. In addition, we used Hegyi’s competition index (HCI) to calculate competitive pressure between live trees and deadwood (prior to death). This index is expressed as: $\text{HCI}=\sum_{j=1}^{n}\frac{d_{j}}{d_{i}}\times \frac{1}{d_{ij}}$ (Equation 6). In this formula, *d*_*j*_ is the DBH of competitor *j*, *d*_*i*_ is the DBH of tree *i*, *d*_*ij*_ is the distance between competitor *j* and tree *i*, and *n* is the number of competitors (Hui et al., 2013; Girona et al., 2019). Using the *deldir* R package, Voronoi diagrams were generated to determine the competitors of object *i* (Turner, 2019). Competitive pressure for each tree was calculated using our own software. Higher values indicate that tree *i* is subject to greater competitive pressure, and vice versa.

## Results

### Quantitative Characteristics of Deadwood

**FIGURE 2:**
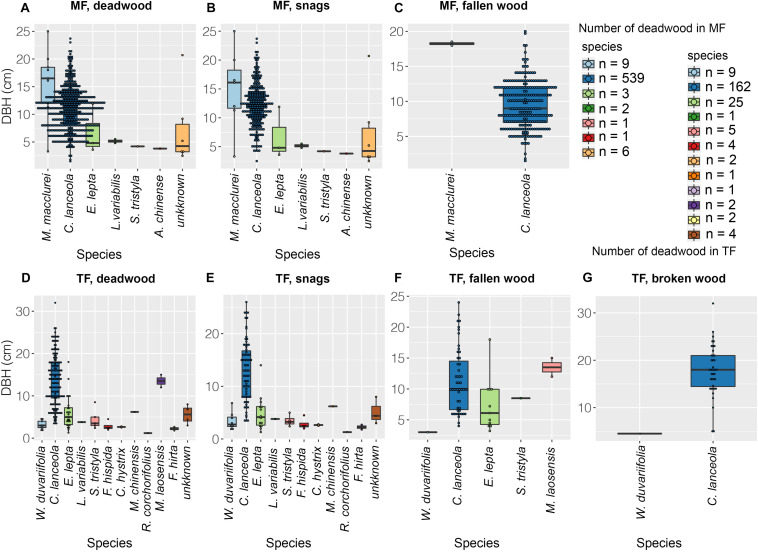
Species and abundance of deadwood. Color indicates species and black dots indicate abundance.

**FIGURE 3:**
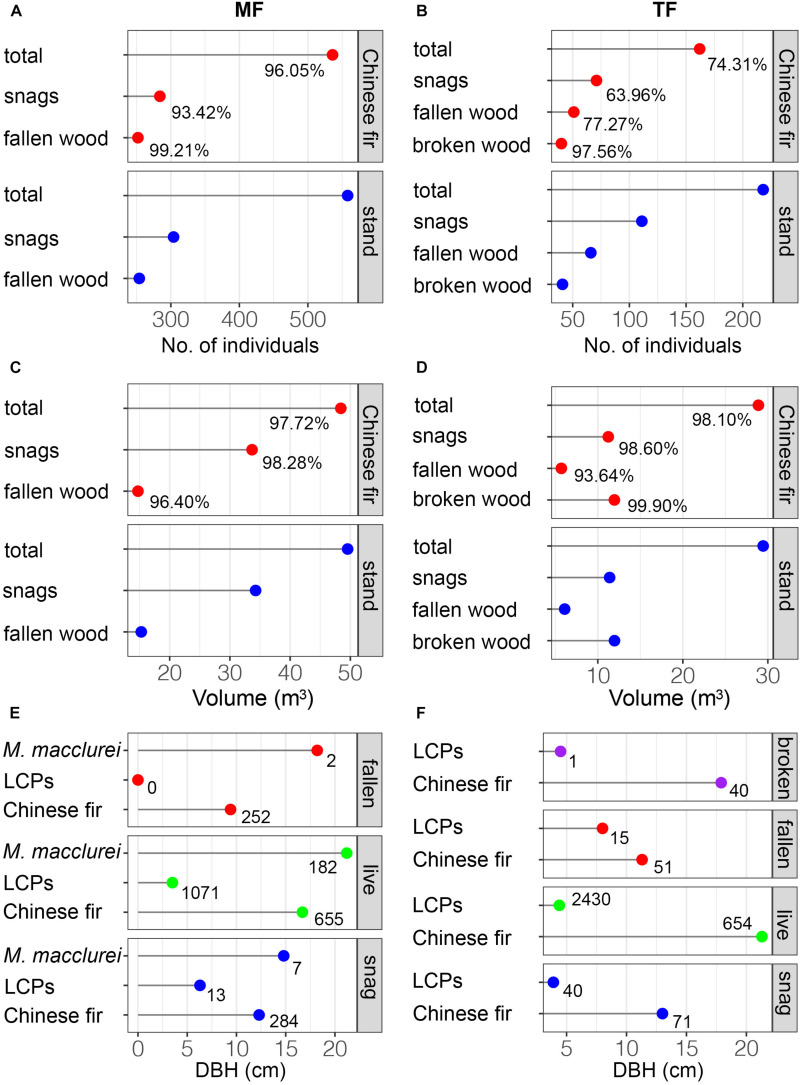
The quantity, volume, and mean DBH of deadwood. The percentage in each panel denotes the proportion of Chinese fir in each stand; integers represent abundance.

Deadwood in the MF consisted of 304 snags and 254 pieces of fallen wood (Figures 2B,C, 3A). Deadwood represented seven species (Figure 2A) and accounted for 22.61% of the total number of stems of the stand (*n* = 2,467). Dead Chinese fir and *M. macclurei* were generally large, although the sizes of individuals varied substantially, whereas individuals of other species were relatively small and less variable in size (Figures 2A–C, 3E). We documented 536 dead Chinese fir trees, representing a 45.0% mortality rate (1,191 trees were originally planted). The TF had 218 dead trees, including 111 snags, 66 pieces of fallen wood, and 41 pieces of broken wood (Figure 3B). Chinese fir accounted for the majority of all types of deadwood, whereas deadwood of the other 14 species was uncommon and small (Figure 2D,G). The total mortality rate in the thinned stand was 6.60%, and the number of Chinese fir after thinning was 162, accounting for 19.86% of the originally planted individuals. In total, 801 Chinese fir trees were lost (162 + 639 stumps), accounting for 55.05% of the originally planted individuals (1993).

In the MF, Chinese fir accounted for 93.42 and 99.21% of snags and fallen wood, respectively, and 96.05% of the stand (Figure 3A). The proportion of Chinese fir in the TF was lower, comprising 63.96% of snags, 77.27% of fallen wood, 97.56% of broken wood, and 74.31% of the stand (Figure 3B). Total volumes of snags and fallen wood in both stands were 48.42, 33.68, 14.73, 28.89, 11.23, and 5.712 m^3^, respectively, of which Chinese fir accounted for the majority: 97.72, 98.28, 96.40 and 98.10, 98.60 and 93.64% (Figures 3C,D). Chinese fir also accounted for 99.90% of the broken wood in the TF (Figure 3D). Fallen wood in the MF comprised 252 Chinese fir with an average DBH of 9.4 cm, and two individuals of *M. macclurei* (Figures 2C, 3E) with an average DBH of 18.2 cm. Snags included seven individuals of *M. macclurei* with an average DBH of 14.8 cm, 13 LCP trees with an average DBH of 6.3 cm, and 284 Chinese fir trees with an average DBH of 12.3 cm (Figure 3E). In the TF, broken wood was represented by 40 Chinese fir trees with a mean DBH of 17.9 cm, and 1 LCP with a DBH of 4.5 cm. Fallen wood in the TF included 51 Chinese fir trees and 15 LCP trees, with mean DBHs of 11.3 cm and 8 cm, respectively, while snags included 71 Chinese fir trees and 40 LCP trees with mean DBHs of 13 and 3.9 cm, respectively (Figure 3F).

### Spatial and DBH Distributions of Deadwood

**FIGURE 4:**
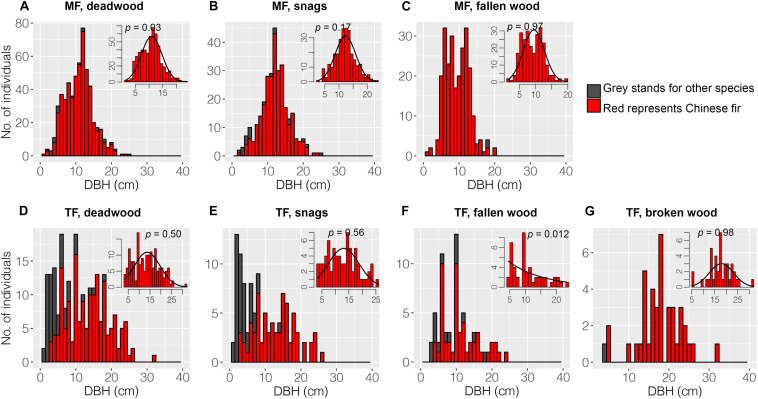
Diameter at breast height distributions of deadwood. Deadwood in the MF consisted of snags and fallen wood, whereas the TF comprised snags, fallen wood, and broken wood. The black curve in each inset panel denotes the DBH distributions for Chinese fir. A *p*-value > 0.05 indicates that the observed data fit the curve.

Chinese fir (red color) dominated the DBH classes of deadwood in the MF, including snags and fallen wood (Figures 4A–C). Other species (gray) were uncommon. Snags were dispersed throughout the DBH classes, whereas fallen wood was only represented in the 18 cm class (Figure 4C). The DBH distributions of snags and fallen wood differed substantially. The Chinese fir showed a normal DBH distribution (Figures 4B,C). In the TF, LCP snags dominated the lower DBH classes; by contrast, LCPs were a minor component of fallen and broken wood (Figures 4E–G). The Chinese fir showed normal or negative exponential DBH distributions in this stand (Figures 4D–G).

**FIGURE 5:**
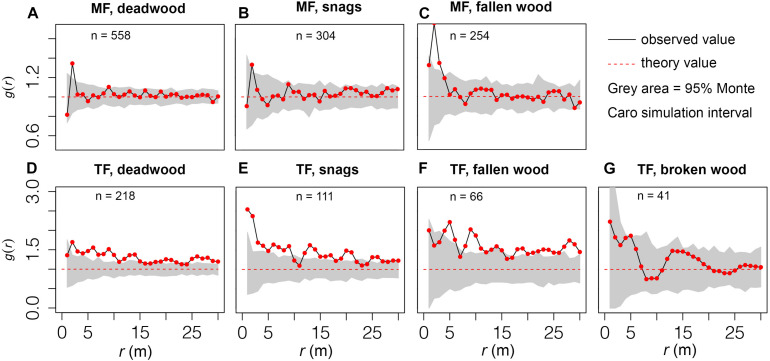
Distribution patterns of deadwood. Observed values located within the gray area denote a random distribution, while values outside of this range are clustered or regular. *n* indicates the abundance of deadwood.

Fallen wood in the MF was clustered at small spatial scales (*r* = 1–3 m) but randomly distributed at other scales (Figures 5A,C), whereas snags exhibited a random distribution pattern at all scales (*r* = 30 m) (Figure 5B). In contrast, deadwood in the TF displayed an aggregated distribution (Figure 5D). In the MCS, the observed values of snags in the TF fell within the ranges of *r* = 10–11 and 22–24 m, but were higher at other scales (Figure 5E). The observed values of fallen wood in the TF were within the ranges of *r* = 1–3 m and 15–17 m, but deviated from these values at other scales (Figure 5F). Broken wood was randomly distributed (*r* = 0–30 m; Figure 5G).

**FIGURE 6:**
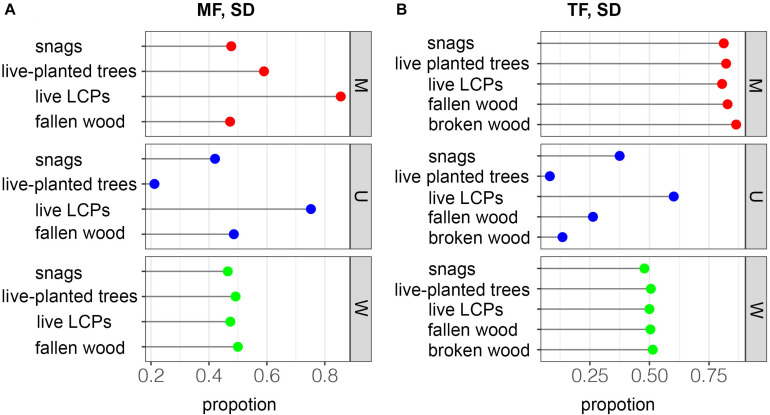
Spatial distributions of deadwood based on the spatial relationships of the four nearest neighbors. *M* = species mixture, *U* = dominance, and *W* = uniform angle index.

In the MF, the *M* value, representing species mixing, was higher in live LCPs (*M* = 0.855) than in live planted trees (0.590), snags (0.477), and fallen wood (0.473). The U value of live LCPs in the MF (0.752) was also higher than that of fallen wood (0.486), snags (0.421), and live planted trees (0.212) (Figure 6A). All groups exhibited a high degree of mixing in the TF, ranging from 0.805 to 0.864; however, dominance varied greatly among groups, and live LCPs (0.602) had a higher U value than snags (0.375), fallen wood (0.263), broken wood (0.135), and live planted trees (0.082) (Figure 6B). All groups exhibited similar W values in both the MF and TF stands (0.465–0.50 and 0.479–0.514) (Figures 6A,B), and were largely within the interval of random distribution (0.475–0.517) reported previously (Li et al., 2020).

### Mortality Rates and Competitive Pressure of Deadwood Before Death

**FIGURE 7:**
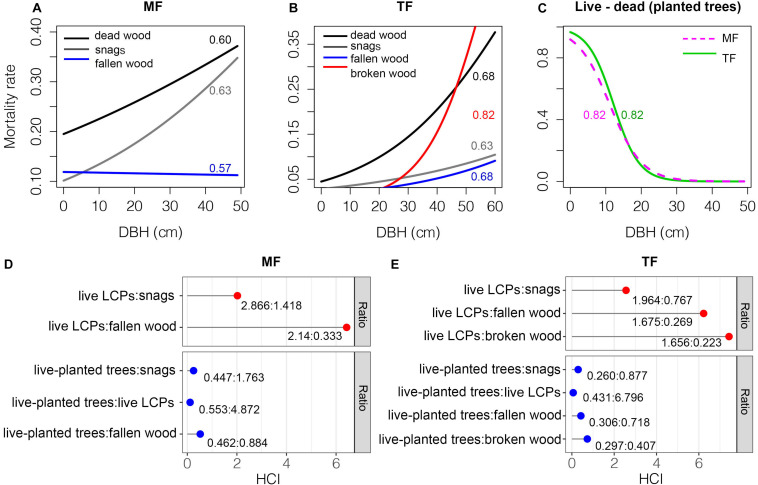
Tree mortality rates **(A–C)** and HCI **(D–E)**. Numbers in panels **(A)–(C)** denote AUC values, and ratios in the lower panels represent mean HCI values by tree group.

Aside from fallen wood in the MF and broken wood in the TF, the mortality rate of trees in both stands increased with increasing DBH. The AUC values were 0.60–0.68 and goodness of fit was thus rated as good in both cases (Figures 7A,B). In contrast, the mortality rates of planted trees decreased rapidly with increasing DBH in both stands (Figure 7C). The AUC value was 0.82 for both stands and goodness of fit was rated as excellent. The competitive pressure of live LCPs in the MF was much higher than that of snags and fallen wood prior to death, with ratios of 2.866:1.418 and 2.14:0.333, respectively (Figure 7D). Live planted trees had a significant competitive advantage over live LCPs and deadwood (Figure 7D). There was a similar competitive relationship in the TF; live LCPs exerted much higher competitive pressure compared to snags, fallen wood, or broken wood before death, but live planted trees had an obvious competitive advantage over LCPs (Figure 7E).

## Discussion

Minor thinning not only allows for early timber harvest, but also promotes the growth of retention trees, prevents invasion by alien species and the development of extreme environmental conditions, ensures the structural stability of stands, and maintains ecological services (Otto et al., 2012; Lu et al., 2018; Hilmers et al., 2020). It is widely used in Chinese fir plantations. The mixture of Chinese fir and *M. macclurei* was selected from among many cultivation practices, and was considered an efficient cultivation method in terms of growth and ecological benefits (Feng et al., 1988; Chen et al., 1990). Li et al. (2020) recently reported that thinning and mixing substantially promote species and SD in Chinese fir plantations at the stand scale. We have further demonstrated that thinning and mixing significantly influence the amount and structure of deadwood in Chinese fir plantations.

### The Abundance of Deadwood

In this study, the abundance of deadwood was much lower in the TF than in the MF (Figures 2, 3), indicating that thinning significantly reduced mortality among planted trees. This is likely attributable to reductions in competition associated with thinning (Figures 7D vs. E), which is consistent with our first hypothesis, as well as with the conventional notion that management activities reduce mortality (Parker et al., 2001; Dittrich et al., 2014; Bölöni et al., 2017; Zhang et al., 2019; Oettel et al., 2020), but contrast to most thinning test in temperature or cool North American (e.g., Carter et al., 2017; Bladon et al., 2008). However, this indicates that thinning removes biomass that could potentially be a component of deadwood, but is instead eliminated in advance (Gronewold et al., 2010; Martin et al., 2014). In contrast, much of the deadwood in the MF comprised Chinese fir of various sizes (Figures 2, 3). Chinese fir, which dominates the MF, is a shade-intolerant species compared with other species and natural stands (Zhang et al., 2017, 2020), and intraspecific competition during the stem exclusion stage is very intense (Figures 7D,E). Kweon and Comeau (2019) reported similar trends in stands of trembling aspen (*Populus tremuloides* Michx.), a fast-growing, shade-intolerant species, and concluded that canopy trees had a higher survival rate than those in the understory. These results suggest that competition results in substantial amounts of deadwood in plantations, although this has scarcely been addressed in the literature. It may also contribute to the promotion and conservation of biodiversity (Thorpe and Thomas, 2007; Dittrich et al., 2014). Outside of instances of severe exogenous disturbance, it is generally accepted that deadwood is abundant in old-growth or virgin forests, although this deadwood originates mainly from senescence rather than competition (Siitonen et al., 2000; Petritan et al., 2012; Kweon and Comeau, 2019).

In this study, the richness and abundance of LCP deadwood was higher in the TF than the MF (Figures 2, 3A,B,E,F). This is because thinning instantaneously changes stand structure and micro-environmental conditions (i.e., light, moisture, and soil temperature), resulting in a large number of species establishing rapidly. Establishment was slower in the MF (Li et al., 2020). With a few exceptions, most studies have concluded that thinning promotes species diversity (Otto et al., 2012). Light is crucial to the survival of seedlings and saplings in the understory (Hurst et al., 2012; Lu et al., 2018). As individuals in the canopy grow and the canopy becomes denser, decreased light levels in the TF may result in the death of some LCPs (Zhu et al., 2010; Zhang et al., 2020). Furthermore, negative density dependence may occur where LCPs are clustered; that is, competition for resources among small conspecifics may result in higher mortality. Negative density dependence is among the most important mechanisms for promoting species co-existence in the early stages of forest succession, and shapes the structure of many forests in tropical, subtropical, and temperate regions (Getzin et al., 2006; Comita and Hubbell, 2009; Gray and He, 2009; Wu et al., 2017). This phenomenon is sometimes referred to as self-thinning (Harvey and Brais, 2007; Nguyen et al., 2016).

Thinning often results in physical damage to retained trees, leading to a specific class of deadwood known as broken wood (Ryall and Smith, 2005; Harvey and Brais, 2007; Chao et al., 2009). Damage to trees caused by thinning has been well-documented, but delayed mortality resulting from thinning remains poorly understood (Thorpe and Thomas, 2007; Bladon et al., 2008; Martin et al., 2014; Monaco et al., 2020). In our study, a small percentage (about 3%) of broken wood was attributable to random thinning of Chinese fir (Figures 2–7). Thinning targets relatively large Chinese fir (DBH ≥ 9 cm), and felling may result in damage to smaller trees, particularly to twigs and upper branches. The red mortality curve implies that the broken wood was, similarly, sized and closely related to live trees (AUC = 0.82, Figure 7B), suggesting that these trees were not killed during thinning; rather, they survived but were ultimately outcompeted by other retained trees (Figure 7E). Many other factors may also lead to breakage (Cremer et al., 1982). In boreal even-aged black spruce [*Picea mariana* (Mill.) B.S.P.] stands where natural disasters were dominated by windthrow, the formation of broken wood was related to small-diameter trunk, rather than silvicultural treatments (Girona et al., 2019). A single instance of broken wood of a non-Chinese fir species (*Wendlandia uvariifolia* Hance) was detected in the TF, and may have been caused by accident (Figure 4G). Trees damaged by thinning encountered greater survival challenges. Seriously injured trees may die quickly; conversely, they may decline gradually as a result of fungal invasion, stain, decay, loss of vitality, or competition (Bladon et al., 2008; Martin et al., 2014). Unfortunately, most studies only present a snapshot of tree mortality (Thorpe and Thomas, 2007); our data also represent only a single point in time. We did not find any broken wood in the MF, which may be attributable to species composition, the relatively small number of individuals in the stand, and the flat terrain in the plot. In short, our results indicate that management methods influence the type and amount of deadwood.

### The Structure of Deadwood

Diameter distribution reflects historical disturbances, site conditions, and developmental processes in stands (Gronewold et al., 2010; Kweon and Comeau, 2019). The diameter distributions of all deadwood types were narrower and had higher kurtosis in the MF compared to the TF, indicating that competition reduces the number of planted trees and increases mortality. Long-term regional studies conducted in southern China have also reported that competition is the primary cause of tree mortality in Chinese fir plantations (Zhang et al., 2017, 2020). In the TF, some of the deadwood before death may acquire increased resource after thinning, whereas trees in the MF remained suppressed. In fact, the vitality of numerous Chinese fir trees in the MF is very low (Li et al., 2020). In contrast, some studies have found that single-tree harvesting narrows the diameter distributions of deadwood (Siitonen et al., 2000; Gronewold et al., 2010). Chinese fir and *M. macclurei* have a mutually facilitative relationship in the early stages of growth (8a) (Feng et al., 1988; Chen et al., 1990). However, the similar mortality curves among the two stands in this study (Figure 7C) suggest that competition is intense among planted trees; this may be related to successional stage (Comita and Hubbell, 2009; Kim and Campbell, 2015).

Distribution patterns of deadwood differed among our study plots. The random distribution of broken wood in the TF is mainly attributable to random harvesting patterns (Figure 5G), and the distribution pattern of damaged trees is strongly influenced by harvesting methods, timeline, stand structure, and engineer experience. Both snags and fallen wood were clustered in the TF (Figures 5E,F), contradicting our third hypothesis. Non-human exogenous disturbances often lead to clumped patterns in terms of tree mortality (Franklin et al., 2007). Microenvironmental conditions, growth status, and changes in the interactions of trees within a stand may trigger asymmetrical competition among them, which could also lead to aggregated mortality. A plausible explanation for our results is that thinning initially promoted the emergence and growth of seedlings and saplings (Parker et al., 2001; Li et al., 2020), whereas canopy closure and negative density dependence later resulted in concentrated mortality. Clustered mortality has been extensively documented in other early successional stands (e.g., Lutz and Halpern, 2006; Iida et al., 2014), and is often considered inevitable (An and MacFarlane, 2013). In contrast, the distribution of mortality in the MF was random or partially random (Figures 5A–C). Based on the small spatial scale of our study (i.e., stand level) we assume that this is not a result of habitat heterogeneity within the plots. Random mixing and uniform planting provide relatively uniform conditions for both colonizing LCPs and interactions among tree species, which may be the main driver of the random distribution. Other studies have reported that randomly distributed deadwood only occurs in old-growth forests or the overstory (e.g., Getzin et al., 2006; Aakala et al., 2012; Petritan et al., 2014). Overall, our results demonstrate that management methods determine the distribution patterns of deadwood at the stand level.

Spatial relationships among nearest neighbors in plantations are relatively simple but are often overlooked in management. However, the structure and composition of Chinese fir plantations become rather complex following conversion (Figures 6A,B). Contrary to our second hypothesis, the M values of deadwood, which indicate the degree of mixture, were much higher in the TF than in the MF (Figures 6A vs. B, in red) and other managed stands and semi-natural forests (Laarmann et al., 2009). Although influenced by distribution patterns, species richness and abundance are often positively correlated with the degree of mixture (e.g., Graz, 2004; Li et al., 2017), as reflected in our results. In the structure unit, live planted trees were dominant, followed by deadwood and live LCPs (Figures 6A,B, in blue); this pattern was also seen in the stands (Figures 7D,E). This suggests a stratified competition, and that suppression is the primary cause of mortality (Lutz and Halpern, 2006). However, the low abundance of LCP deadwood and large number of live LCP trees imply that competition calculated based on distances between trees and tree sizes does not accurately reflect competitive pressure (Figures 3E,F vs. 7D,E). Hurst et al. (2012) held a similar view. The mortality curve also reflects vertical changes in competition (Figure 7A,B). Along with successional stage, some studies have reported that species traits (e.g., shade tolerance and architecture) are important in analyses of competition, especially in uneven-aged MFs (Pommerening and Meador, 2018). Small trees do not necessarily experience high mortality rates, and vice versa.

## Conclusion

The conversion of natural forests to plantations has been widely studied, but the findings of these studies cannot be applied to the conversion of plantations to uneven-aged MFs, resulting in an absence of suitable guidelines. For sustainable forest management, the issue of timber supply must be resolved; forest biodiversity, ecological functions and benefits, and social services must also be attended to, which requires a change in the silvicultural methods used in plantations. The challenge is to transform low-yield and low-quality artificial forests into uneven-aged MFs, or to cultivate MFs from scratch. The amount, structure and type of deadwood in Chinese fir plantations implies that thinning and mixing result in different stand development processes. In this study, compared to the mixed stand, thinning reduced the density and mortality rates of planted trees, as well as the number and volume of dead trees. Although some underlying mechanisms of plantations and natural forests (e.g., root grafts) maybe beneficial to tree growth (Jelínková et al., 2009; Tarroux and DesRochers, 2011), a small part of retained planted trees was eliminated via long-term competition, highlighting the necessity of thinning. Thinning also significantly increased species richness and abundance, suggesting that reducing competition by planting species over a long time scale could be advantageous. The two study stands are in the early stages of succession, and competition between planted trees is still the most important driver of mortality; this suggests that reducing competition may be the key to successful cultivation of Chinese fir MFs. Thinning could be the best method that reduces neighbor competition in direct. We suggest prolonging rotation period and converting pure Chinese fir plantations into MFs by slight thinning. The elimination of the broken wood at 10 years requires us to minimize the damage to the retained trees.

The large amount of deadwood in the MF may provide sufficient habitat resources and favors non-woody organisms. Planting, and subsequently thinning, mixed Chinese fir forests may thus be an ecologically appropriate silvicultural method in future. The reasons for tree mortality in Chinese fir plantations are clear on the whole, except for some uncounted tree-level factors, such as dead time, plant diseases, insect pests and senescence. More studies should be conducted to assess the relationship between deadwood and biodiversity, wildlife and to support conversion planning at regional and global scales.

## Data Availability Statement

The raw data supporting the conclusions of this article will be made available by the authors, without undue reservation.

## Author Contributions

YuL collected the data and drafted the manuscript. SY revised the manuscript and participated in analyzing the experiment data. MuL, XL, ZL, AM, and HL participated in collecting the experimental data. All authors contributed to the article and approved the submitted version.

## Conflict of Interest

The authors declare that the research was conducted in the absence of any commercial or financial relationships that could be construed as a potential conflict of interest.
